# Comparative Diagnostic Performance of Amyloid‐β Positron Emission Tomography and Magnetic Resonance Imaging in Alzheimer's Disease: A Head‐to‐Head Meta‐Analysis

**DOI:** 10.1002/brb3.70111

**Published:** 2024-10-22

**Authors:** Fang Li, Jiang Cheng, Kaihui Jin, Li Zhao, Junyong Li, Jia Wu, Xiaolu Ren

**Affiliations:** ^1^ Department of Neurology General Hospital of Ningxia Medical University Yinchuan China; ^2^ Department of Radiology General Hospital of Ningxia Medical University Yinchuan China; ^3^ School of Health Sciences Universiti Sains Malaysia Kubang Kerian Kelantan Malaysia

**Keywords:** Alzheimer's disease, amyloid‐β, magnetic resonance imaging, positron emission tomography

## Abstract

**Objective:**

This meta‐analysis aimed to evaluate the comparative diagnostic performance of amyloid‐β positron emission tomography (Aβ PET) and magnetic resonance imaging (MRI) in diagnosing Alzheimer's disease (AD).

**Methods:**

An extensive search was conducted in the PubMed and Embase databases to identify available publications up to December 2023. Head‐to‐head comparative studies were included if they evaluated the diagnostic performance of Aβ PET and MRI in diagnosing Alzheimer's disease. Sensitivity and specificity were assessed using the DerSimonian and Laird method, followed by transformation via the Freeman–Tukey double inverse sine transformation.

**Results:**

Six articles involving 560 patients were included in the meta‐analysis. When distinguishing AD from mild cognitive impairment (MCI), both methods showed comparable sensitivity (Aβ PET: 0.71, MRI: 0.62) and specificity (Aβ PET: 0.68, MRI: 0.69), with no statistically significant differences observed (*p* = 0.34 and 0.99). When identifying AD from normal cognitive control (NC), both Aβ PET and MRI showed similar results, with comparable sensitivity (Aβ PET: 0.93, MRI: 0.85) and specificity (Aβ PET: 0.95, MRI: 0.82), without significant differences (*p* = 0.38 and 0.19). Similarly, in detecting MCI from NC, both Aβ PET and MRI demonstrated similar sensitivity (Aβ PET: 0.69, MRI: 0.64) and specificity (Aβ PET: 0.75, MRI: 0.76) without significant differences (*p* = 0.40 and 0.94). However, 18F‐FMM seems to have a higher specificity compared to MRI when distinguishing AD from MCI (*P* = 0.03) and AD from NC (*p* = 0.04).

**Conclusions:**

Our meta‐analysis indicates that Aβ PET demonstrates similar sensitivity and specificity to MRI in diagnosing Alzheimer's disease. However, the limited number of studies may impact the evidence of the current study; further larger sample prospective research is required to confirm these findings.

## Graphical abstract

This meta‐analysis compares the diagnostic performance of amyloid‐β PET and MRI in Alzheimer's disease. Both imaging methods demonstrate similar sensitivity and specificity in distinguishing AD from MCI and NC. However, 18F‐FMM shows higher specificity in certain diagnostic comparisons.

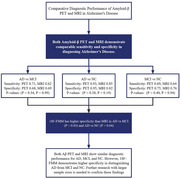

## 1 Introduction

Alzheimer's disease (AD) is a progressively advancing neurodegenerative disorder and the most prevalent form of dementia, affecting millions globally (Alzheimer's Association 2023). It is primarily characterized by cognitive decline and memory impairment (Seo and Holtzman 2024). Mild cognitive impairment (MCI) is often considered an early stage of AD, presenting with less severe symptoms (Jongsiriyanyong and Limpawattana 2018). Early diagnosis is vital for slowing disease progression, enhancing quality of life, and providing better planning and support for affected individuals and their families (Pais et al. 2020). Therefore, accurately differentiating MCI, AD, and normal cognitive control (NC) is a key area of medical research.

Traditionally, the definitive diagnosis of AD has been reliant on postmortem brain tissue pathology, which is impractical and unethical to perform on living patients (Porsteinsson et al. 2021; DeTure and Dickson 2019). This method detects the signature amyloid plaques and neurofibrillary tangles in brain tissue, which are the distinctive hallmarks of AD in living patients (King, Bodi, and Troakes 2020). Single photon emission computed tomography (SPECT) has also been employed to indicate the presence of changes related to AD (Bloudek et al. 2011). However, brain biopsies pose risks of tissue damage and exacerbation of cognitive impairment, making them unsuitable for routine diagnostic use (Porsteinsson et al. 2021; Breijyeh and Karaman 2020). SPECT, while noninvasive, often lacks the specificity and sensitivity needed for early and accurate diagnosis of AD (Bloudek et al. 2011).

Fluorodeoxyglucose positron emission tomography (FDG PET) is commonly used to assess glucose metabolism in the brain, providing insights into neuronal activity and synaptic function (Mosconi 2005). FDG PET can detect reduced glucose metabolism in areas like the temporoparietal cortex and posterior cingulate, which reflects neurodegeneration and is useful in identifying the early stages of AD (Chételat et al. 2020). However, it lacks specificity for AD because reduced glucose metabolism occurs in other neurodegenerative conditions like frontotemporal dementia (Chételat et al. 2020). In recent years, the use of amyloid‐β PET (Aβ PET) and magnetic resonance imaging (MRI) in the diagnosis of AD has become increasingly prevalent. Aβ PET, particularly with tracers like carbon‐11 labeled Pittsburgh compound B (11C‐PIB), 18F‐florbetapir (18F‐AV45), and 18F‐flutemetamol (18F‐FMM), is highly effective in targeting amyloid plaques in the brain, which are a key pathological feature of AD (Ruan and Sun 2023; Morris et al. 2016). The tracer 11C‐PIB is known for its strong correlation with neuroinflammatory plaques and vascular amyloid, as shown in autopsies (Ikonomovic et al. 2008). The 18F‐labeled tracers, such as 18F‐AV45, approved by the FDA in 2011 (Yang, Rieves, and Ganley 2012), and 18F‐FMM, a derivative of 11C‐PIB with similar uptake in the cerebral cortex (Nelissen et al. 2009), offer advantages over earlier tracers, particularly in overcoming their nontechnical limitations.

MRI, now a widely common and accessible tool, offers detailed brain structure imaging and is crucial for identifying characteristic patterns of brain atrophy in AD (Frisoni et al. 2010). Despite their exceptional performance, a debate persists regarding their relative diagnostic effectiveness (Kitajima et al. 2021; Wang et al. 2016). This controversy arises due to the lack of head‐to‐head comparisons, leading to uncertainties among clinicians and researchers about the most effective imaging technique for AD diagnosis.

The purpose of this meta‐analysis is to compare the diagnostic performance of amyloid‐β PET and MRI in Alzheimer's disease by head‐to‐head comparison.

## 2 Methods

In conducting the meta‐analysis, we adhered strictly to the Preferred Reporting Items for Systematic Reviews and Meta‐Analyses of Diagnostic Test Accuracy (PRISMA‐DTA) guidelines (McInnes et al. 2018).

### 2.1 Search Strategy

A comprehensive literature search was executed across the PubMed and Embase databases, capturing publications available up to December 2023. This search employed specific keywords: “Alzheimer's disease,” “amyloid,” “positron‐emission tomography,” and “magnetic resonance imaging.” Further details are presented in Table S1. The reference lists of the selected studies were also thoroughly searched to identify additional relevant articles.

### 2.2 Inclusion and Exclusion Criteria

Inclusion Criteria: (1) Studies must be either retrospective or prospective head‐to‐head comparative analyses; (2) focus on evaluating the diagnostic performance of Aβ PET and MRI for Alzheimer's disease diagnosis; (3) one of the following outcomes were included: AD vs. MCI, AD vs. NC, MCI vs. NC; (4) minimum sample size: more than 10 patients.

Exclusion Criteria: (1) Duplicate articles, abstracts without full texts, editorial comments, letters, case reports, reviews, and meta‐analyses; (2) studies with irrelevant titles or abstracts; (3) non‐English full‐text articles; (4) studies with incomplete or unclear data necessary for calculating the sensitivity or specificity of the imaging modality.

### 2.3 Quality Assessment

Two researchers independently evaluated the quality of the included studies using the Quality Assessment of Diagnostic Accuracy Studies‐2 (QUADAS‐2) tool (Whiting et al. 2011). This tool covers four key domains: (1) patient selection, (2) index test, (3) reference standard, and (4) flow and timing. Each study's risk of bias was categorized as either “high risk,” “low risk,” or “unclear risk.”

### 2.4 Data Extraction

Two researchers independently extracted data from the included articles. This data encompassed details such as author, publication year, and the specific imaging test employed in each study. Additionally, study characteristics such as country, design, period, reference standard, and image analysis were extracted, along with patient characteristics like patient count, type, mean or median age, and gender. In cases of disagreement, the researchers engaged in discussion to reach consensus, thereby ensuring the accuracy of the extracted data.

### 2.5 Statistical Analysis

We assessed sensitivity and specificity using the DerSimonian and Laird method, followed by transformation through the Freeman–Tukey double inverse sine transformation. We employed the Jackson method to calculate confidence intervals. Heterogeneity within and between groups was evaluated using the Cochrane Q and *I* ^2^ statistics. In cases where there was high heterogeneity between the studies (*I^2^* > 50%), we conducted leave‐one‐out sensitivity analysis to identify the source of this heterogeneity. In addition, we performed subgroup analyses based on different radiopharmaceutical types, including 11C‐PIB, 18F‐FMM, and 18F‐AV45.

To evaluate publication bias, we utilized both funnel plots and Egger's test. Statistical significance was defined as *p* < 0.05. All statistical analyses were performed using R software version 4.1.4 for computing and generating graphics.

## 3 Results

### 3.1 Study Selection

**FIGURE 1:**
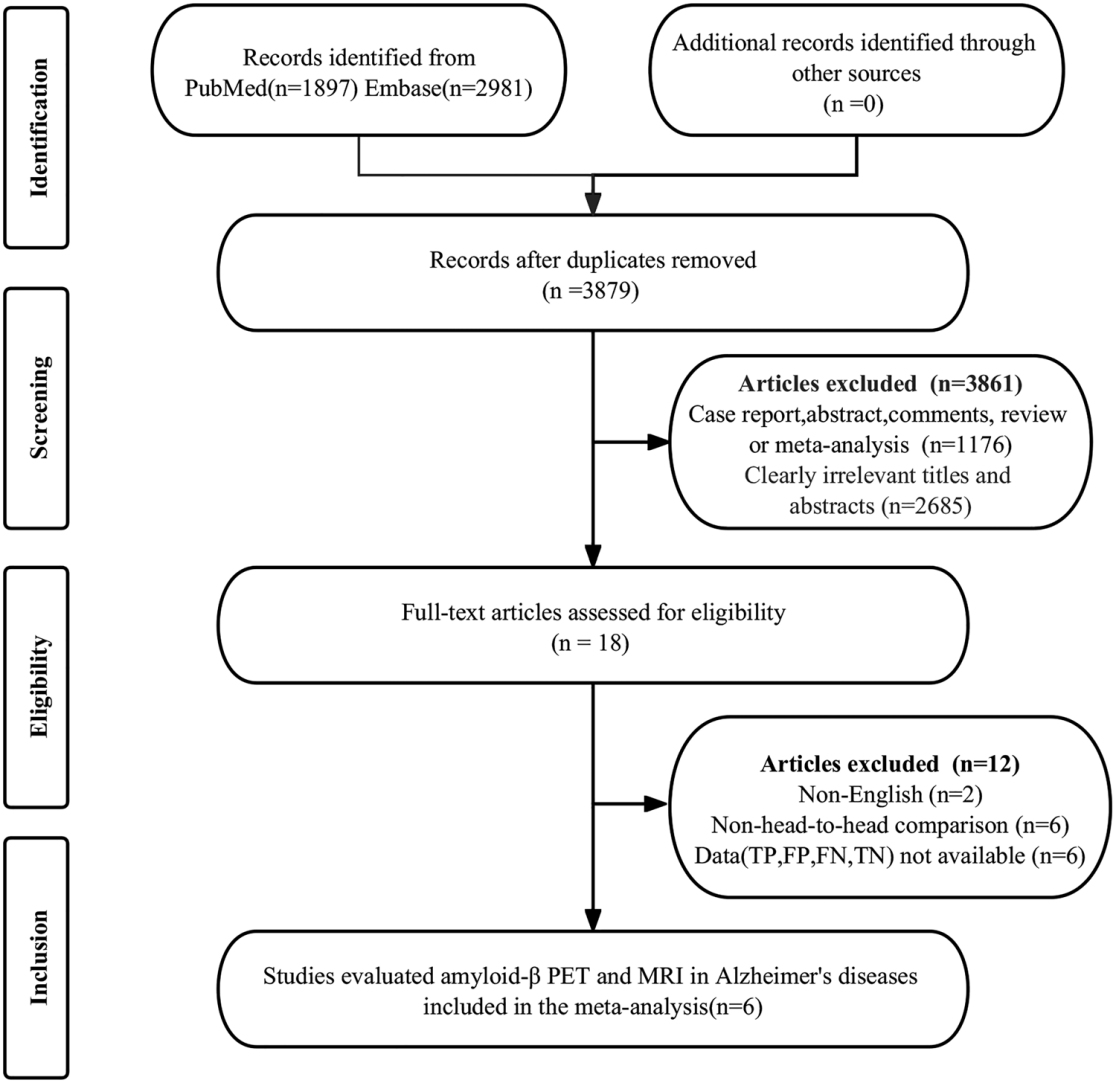
PRISMA flow diagram illustrating the study selection process.

The preliminary search revealed a total of 4878 publications. However, 999 studies were considered duplicates, and another 3861 did not meet the eligibility criteria and were therefore not included in the study. After a comprehensive review of the full texts of the remaining 18 articles, another 10 were deemed ineligible for the study because data (TP, FP, FN, and TN) were not available (*n* = 6). In addition, non‐English articles (*n* = 2) and Aβ PET without directly being compared to MRI articles (*n* = 6) were excluded. Finally, six articles evaluating the comparative diagnostic efficacy of Aβ PET and MRI in diagnosing Alzheimer's disease were included in the meta‐analysis (Kitajima et al. 2021; Wang et al. 2016; Mikhno et al. 2012; Trzepacz et al. 2014; Vandenberghe et al. 2013; Xu et al. 2016). The article selection process, according to the PRISMA flow diagram, is depicted in Figure 1.

### 3.2 Study Description and Quality Assessment

The six eligible studies included a total of 560 patients (range from 26 to 227). Among the included studies, two articles were retrospective studies, while four articles were prospective studies. Two studies used 18F‐AV45, one study used 18F‐FMM, and three studies used 11C‐PIB for amyloid imaging. Two studies using 18F‐Florbetaben (18F‐FBB) did not meet our inclusion criteria for the meta‐analysis (Villemagne et al. 2012; Yoon et al. 2021). Two articles did not provide information about reference standard and four articles solely relied on follow‐up imaging as the reference standard. Five of the six studies used semiquantitative methods for both PET and MRI analyses, while one study used visual analysis for both modalities. Table 1 summarizes the study and patient characteristics of the included studies. Table 2 provides a detailed overview of the diagnostic criteria used in the included studies for AD and MCI. Most studies followed the NINCDS‐ADRDA criteria for AD diagnosis and Petersen's criteria for MCI, with some variation in additional neuropsychological assessments. In the included studies, MRI was mainly used to measure brain atrophy, particularly in the hippocampus and temporal lobes. Kitajima et al. (2021) and Mikhno et al. (2012) focused on hippocampal atrophy. Trzepacz et al. (2014) examined atrophy in the hippocampus and temporal regions to predict MCI progression. Vandenberghe et al. (2013) and Wang et al. (2016) assessed gray matter volumes, while Xu et al. (2016) measured hippocampal and amygdala volumes to predict MCI conversion to AD.

**TABLE 1:** Study and patient characteristics of the included studies.

			Study characteristics					Patient characteristics			
Author	Year	Type of imaging test	Country	Study design	Study period	Reference standard	Image analysis	No. of patients	Types of patients	Mean/Median age	Male/Female
Kitajima et al.	2021	11C‐PIB PET vs. MRI	Japan	Pro	2018‐2020	NA	Visual	26	AD(7), NC(4), MCI(15)	Mean ± SD:(78.5 ± 5.18)	5/21
Wang et al.	2016	18F‐AV 45 PET vs. MRI	China	Retro	NA	Follow‐up	Semi‐quantitative	129	AD(64), MCI (65)	Mean ± SD: AD: 72.5 ± 7.4 MCI: 72.2 ± 7.5	74/55
Xu et al.	2016	18F‐AV 45 PET vs. MRI	China	Retro	NA	Follow‐up	Semi‐quantitative	227	AD(27), NC(117), MCI(83)	Mean ± SD: AD,74.0 ± 7.6 MCI,75.7 ± 7.9 NC, 75.4 ± 7.0	121/106
Trzepacz et al.	2014	11C‐PIB PET vs. MRI	USA	Pro	NA	Follow‐up	Semi‐quantitative	50	AD(20), MCI(30)	Mean ± SD: AD, 75.4 ± 6.6 MCI, 74.2 ± 8.4	33/17
Vandenberghe et al.	2013	18F‐FMM PET vs. MRI	Belgium	Pro	NA	Follow‐up	Semi‐quantitative	72	AD(27), NC(25), MCI(20)	Mean ± SD: AD, 69.9 ± 7.03 MCI, 72.7 ± 7.09 NC,56.38 ± 17.90	NA
Mikhno et al.	2012	11C‐PIB PET vs. MRI	USA	Pro	NA	NA	Semi‐quantitative	56	AD, 17, NC(17), MCI(22)	NA	NA

Abbreviations: AD, Alzheimer's disease; NC, normal cognitive control; MCI, mild cognitive impairment;18F‐AV45, 18F‐florbetapir; 18F‐FMM, 18F‐flutemetamol; Pro, prospective; Retro, retrospective; NA, not available.

**TABLE 2:** Detailed Alzheimer's disease and mild cognitive impairment diagnostic criteria.

Author, year	AD diagnostic criteria	MCI diagnostic criteria
Kitajima et al. (2021)	NINCDS‐ADRDA criteria	Not explicitly mentioned
Mikhno et al. (2012)	NINCDS‐ADRDA criteria	Petersen's criteria: memory impairment with MMSE scores in the range of 24–30, normal daily functioning
Trzepacz et al. (2014)	NINCDS‐ADRDA, DSM‐IV for Alzheimer's dementia	Petersen's criteria: memory impairment, CDR of 0.5, MMSE score 24–30, no significant impairment in other domains
Vandenberghe et al. (2013)	NINCDS‐ADRDA, DSM‐IV for Alzheimer's dementia	Petersen's criteria: amnestic MCI, CDR of 0.5, memory loss documented through neuropsychological testing
Wang et al. (2016)	No specific AD diagnosis criteria	Subjective memory complaint, objective memory loss (Wechsler Memory Scale), MMSE 24–30, CDR 0.5
Xu et al. (2016)	No specific AD diagnosis criteria	MMSE 24‐30, CDR 0.5, subjective memory complaint, objective memory loss (Wechsler Memory Scale)

Abbreviations: AD, Alzheimer's disease; MCI, mild cognitive impairment; NINCDS‐ADRDA, National Institute of Neurological and Communicative Disorders and Stroke—Alzheimer's Disease and Related Disorders Association; DSM‐IV: Diagnostic and Statistical Manual of Mental Disorders, 4th Edition; MMSE, Mini‐Mental State Examination; CDR, clinical dementia rating.

**FIGURE 2:**
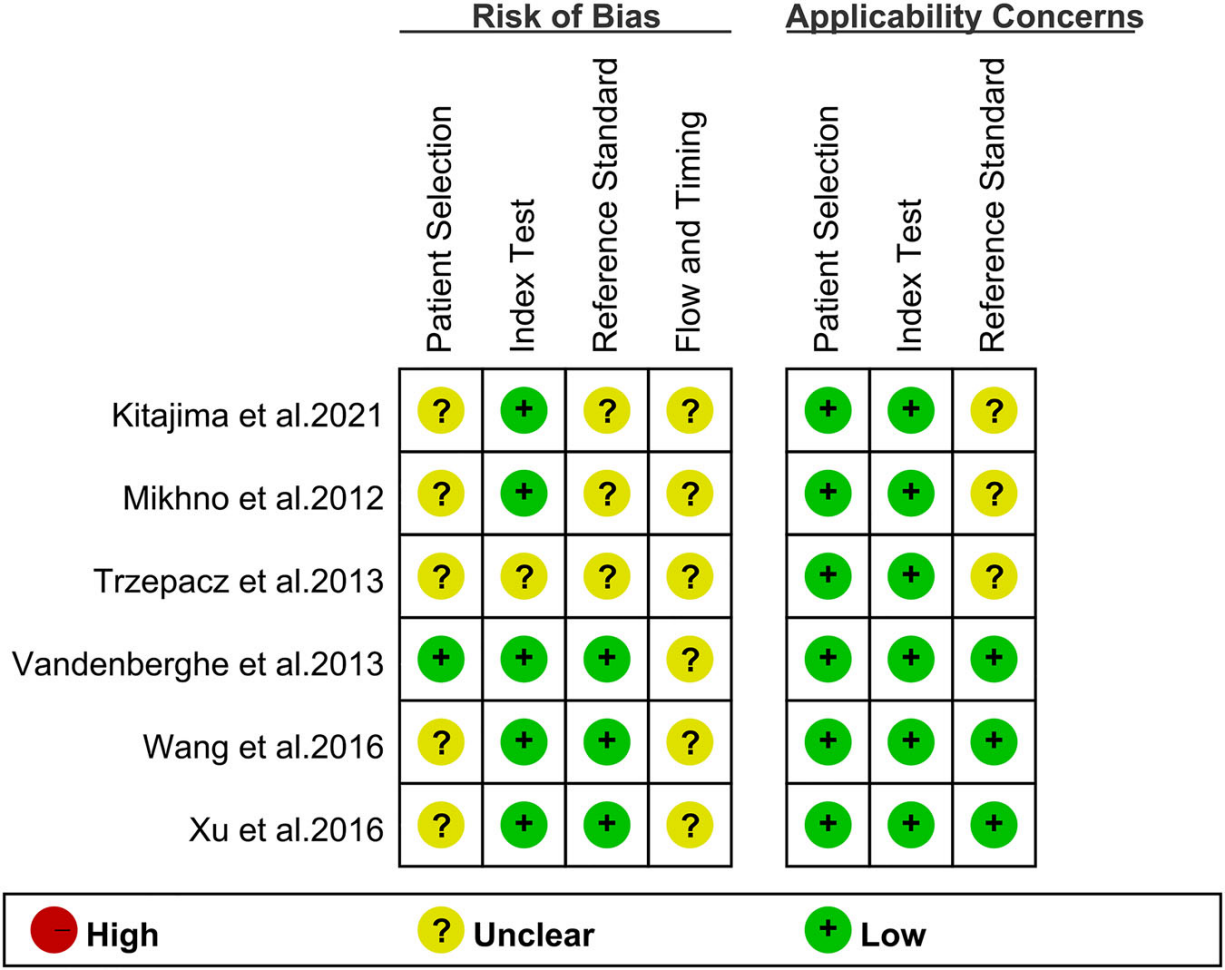
Assessment of bias and applicability concerns in included studies using the QUADAS‐2 tool. QUADAS‐2, Quality Assessment of Diagnostic Accuracy Studies‐2.

The assessment of bias risk for each study, using the QUADAS‐2 tool, is presented in Figure 2. For the patient selection risk of bias assessment, we found five studies graded as “unclear risk” due to their failure to provide information regarding the inclusion of consecutive patients. For the index test, one study received an “unclear risk” rating, as it remained unclear whether the cut‐off values had been predetermined. For the reference standard, three studies were categorized as “unclear,” as they lacked information confirming independent determination by two or more physicians for the final diagnosis. The evaluation of flow and timing standards resulted in an “unclear risk” rating for all six studies due to the lack of clarity regarding the time interval between the diagnostic test and the reference standard. Overall, the quality assessment did not reveal major concerns regarding the included studies.

### 3.3 Comparing the Sensitivity of Aβ PET and MRI in Diagnosing Alzheimer's Disease

**FIGURE 3:**
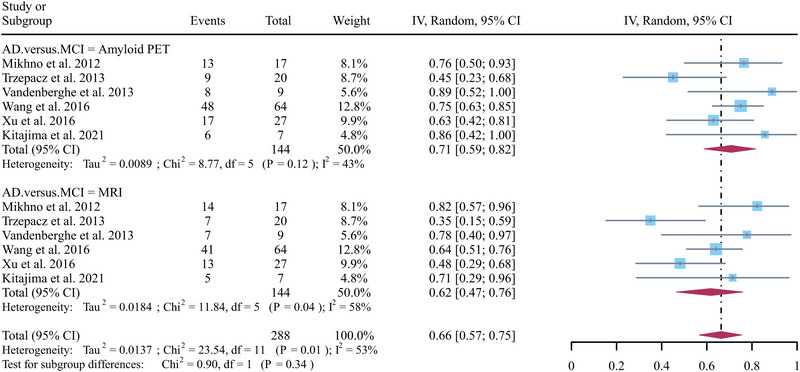
Forest plot demonstrating pooled sensitivity of Aβ PET and MRI in detecting AD from MCI. AD, Alzheimer's disease; MCI, mild cognitive impairment.

**FIGURE 4:**
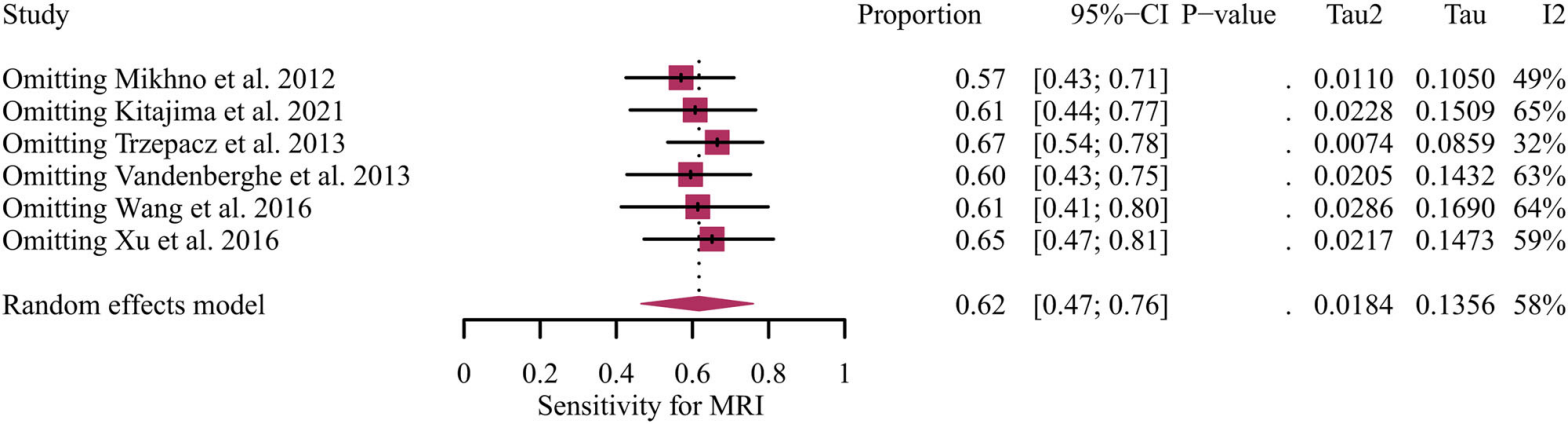
Leave‐one‐out sensitivity analysis for MRI's sensitivity in detecting AD from MCI. AD, Alzheimer's disease; MCI, mild cognitive impairment.

A total of six studies focusing on AD versus MCI were included in the analysis, and the pooled sensitivity of Aβ PET and MRI in detecting AD from MCI were 0.71 (95% CI: 0.59–0.82) and 0.62 (95% CI: 0.47–0.76) (Figure 3). There was no significant difference between Aβ PET and MRI in the sensitivity for distinguishing AD from MCI (*p* = 0.34) (Figure 3). Regarding the pooled overall sensitivity of MRI, the *I^2^* was 58%. The leave‐one‐out sensitivity analysis revealed that removing Trzepacz et al.’s (2014) study reduced heterogeneity (*I^2^* = 32%), while removing Mikhno et al.’s (2012) study had a similar effect on MRI (*I^2^* = 49%) (Figure 4).

A total of three studies focusing on AD versus NC were included in the analysis, and the pooled sensitivity of Aβ PET and MRI in detecting AD from NC were 0.93 (95% CI: 0.77–1.00) and 0.85 (95% CI: 0.73–0.95) (Figure S1). There was no significant difference between Aβ PET and MRI in the sensitivity for distinguishing AD from NC (*p* = 0.38).

A total of three studies focusing on MCI versus NC were included in the analysis, and the pooled sensitivity of Aβ PET and MRI in detecting MCI from NC were 0.69 (95% CI: 0.61–0.77) and 0.64 (95% CI: 0.56–0.72) (Figure S2). There was no significant difference between Aβ PET and MRI in the sensitivity for distinguishing MCI from NC (*p* = 0.40).

### 3.4 Comparing the Specificity of Aβ PET and MRI in Diagnosing Alzheimer's Disease

**FIGURE 5:**
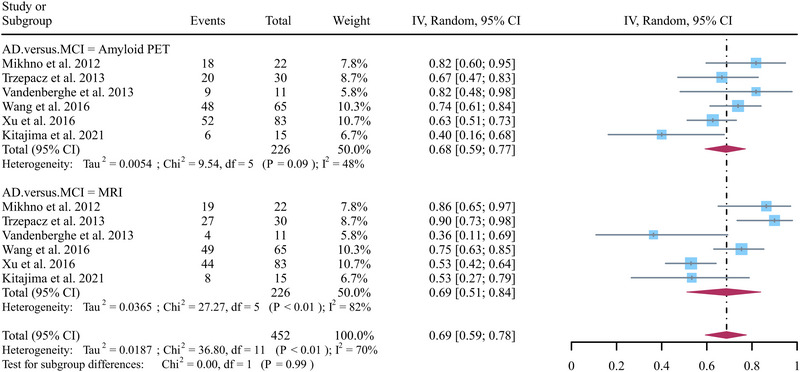
Forest plot illustrating the pooled specificity of Aβ PET and MRI in detecting AD from MCI. AD, Alzheimer's disease; MCI, mild cognitive impairment.

**FIGURE 6:**
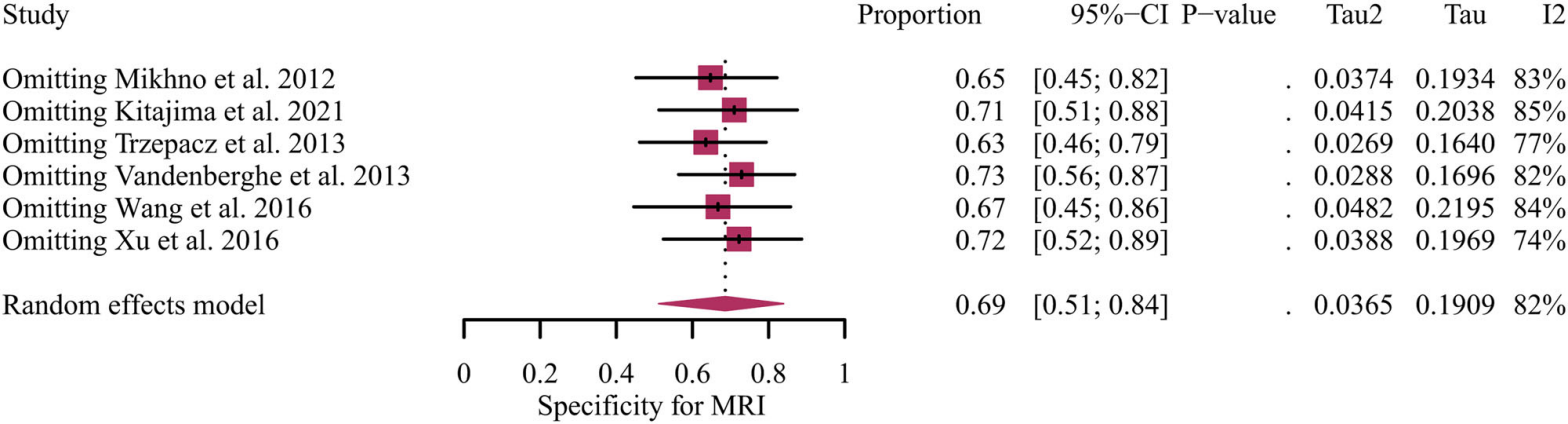
Leave‐one‐out specificity analysis for MRI in detecting AD from MCI. AD, Alzheimer's disease; MCI, mild cognitive impairment.

A total of six studies focusing on AD versus MCI were included in the analysis, and the pooled specificity of Aβ PET and MRI in detecting AD from MCI were 0.68 (95% CI: 0.59–0.77) and 0.69 (95% CI: 0.51–0.84) (Figure 5). There was no significant difference between Aβ PET and MRI in the specificity for distinguishing AD from MCI (*p* = 0.99) (Figure 5). Regarding the pooled overall specificity of MRI, the *I* ^2^ was 82%. The leave‐one‐out sensitivity analysis found no source of heterogeneity (Figure 6).

A total of three studies focusing on AD versus NC were included in the analysis, and the pooled specificity of Aβ PET and MRI in detecting AD from NC were 0.95 (95% CI: 0.85–1.00) and 0.82 (95% CI: 0.58–0.98) (Figure 3). There was no significant difference between Aβ PET and MRI in the specificity for distinguishing AD from NC (*p* = 0.19).

A total of three studies focusing on MCI versus NC were included in the analysis, and the pooled specificity of Aβ PET and MRI in detecting MCI from NC were 0.75 (95% CI: 0.67–0.83) and 0.76 (95% CI: 0.67–0.83) (Figure 4). There was no significant difference between Aβ PET and MRI in the specificity for distinguishing MCI from NC (*p* = 0.94).

### 3.5 Comparing the Sensitivity and Specificity of Subgroup Aβ PET and MRI in Diagnosing Alzheimer's Disease

For 11C‐PIB subgroup, there were no significant difference between 11C‐PIB and MRI in the sensitivity and specificity when distinguishing AD from MCI (*p* = 0.82, 0.35), AD from NC (*p* = 0.31, 0.64), and MCI from NC (*p* = 0.63, 0.18) (Table 3).

**TABLE 3:** Subgroup analysis based on specific radiotracer in head‐to‐head comparison between amyloid‐β PET and MRI in Diagnosis of Alzheimer's disease.

	Amyloid‐β PET				MRI				*P* value between amyloid‐β PET and MRI (*P* < 0.05 was consider significant difference)
Outcome measure	No. of studies	No. of total patients	No. of total events	Pooled results (95% CI)	No. of studies	No. of total patients	No. of total events	Pooled results (95% CI)	
**11C‐PIB**									
AD versus MCI (Sensitivity)	3	44	28	0.67 (0.42–0.89)	3	44	26	0.63 (0.31–0.90)	0.82
AD versus MCI (Specificity)	3	67	44	0.65 (0.41–0.85)	3	67	54	0.79 (0.56–0.96)	0.35
AD versus NC (Sensitivity)	2	24	23	0.97 (0.74–1.00)	2	24	20	0.85 (0.66–0.98)	0.31
AD versus NC (Specificity)	2	21	20	0.97 (0.82–1.00)	2	21	19	0.93 (0.75–1.00)	0.64
MCI versus NC (Sensitivity)	2	37	27	0.73 (0.49–0.91)	2	37	24	0.64 (0.32–0.90)	0.63
MCI versus NC (Specificity)	2	21	15	0.82 (0.39–1.00)	2	21	16	0.78 (0.55–0.95)	0.18
**18F‐AV45**									
AD versus MCI (Sensitivity)	2	91	65	0.72 (0.62–0.81)	2	91	54	0.59 (0.49–0.70)	0.09
AD versus MCI (Specificity)	2	148	100	0.68 (0.57–0.78)	2	148	93	0.64 (0.42–0.84)	0.77
MCI versus NC (Sensitivity)	1	117	86	0.74 (0.65–0.81)	2	117	86	0.74 (0.65–0.81)	1.00
MCI versus NC (Specificity)	1	110	74	0.67 (0.58–0.76)	2	110	70	0.64 (0.54–0.73)	0.57
**18F‐FMM**									
AD versus MCI (Sensitivity)	1	9	8	0.89 (0.52–1.00)	1	9	7	0.78 (0.40–0.97)	0.57
AD versus MCI (Specificity)	1	11	9	0.82 (0.48–0.98)	1	11	4	0.36 (0.11–0.69)	**0.03**
AD versus NC (Sensitivity)	1	27	23	0.85 (0.66–0.96)	1	27	23	0.85(0.66‐0.96)	1.00
AD versus NC (Specificity)	1	25	23	0.92 (0.74–0.99)	1	25	17	0.68 (0.46–0.85)	**0.04**

Abbreviations: **11C‐PIB,** carbon‐11 labeled Pittsburgh compound B**;** 18F‐AV45, 18F‐florbetapir; 18F‐FMM, 18F‐flutemetamol; AD, Alzheimer's disease; MCI, mild cognitive impairment; NC normal cognitive control.

For 18F‐AV45 subgroup, there were no significant difference between 18F‐AV45 and MRI in the sensitivity and specificity when distinguishing AD from MCI (*p* = 0.09, 0.77) and MCI from NC (*p* = 1.00, 0.57) (Table 3).

For 18F‐FMM subgroup, there were no significant difference between 18F‐FMM and MRI in the sensitivity when distinguishing AD from MCI (*p* = 0.57) and AD from NC (*p* = 1.00) (Table 3). However, 18F‐FMM seems to have a higher specificity compared to MRI when distinguishing AD from MCI (*p* = 0.03) and AD from NC (*p* = 0.04) (Table 3).

### 3.6 Publication Bias of Aβ PET and MRI in Diagnosing Alzheimer's Disease

**FIGURE 7:**
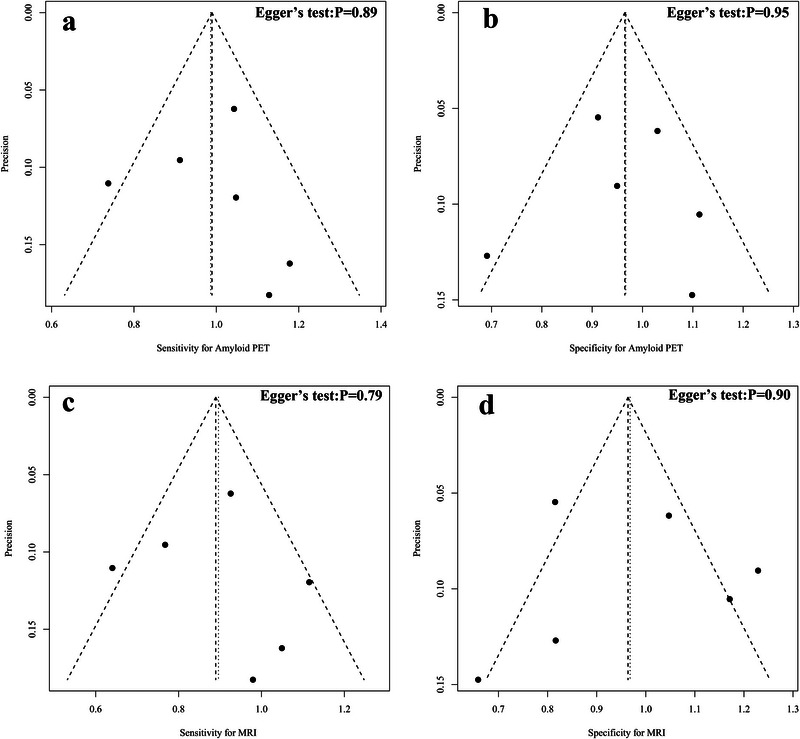
Funnel plot and Egger's test for (a) Aβ PET sensitivity; (b) Aβ PET MRI specificity; (c) MRI sensitivity; (d) MRI specificity.

The funnel plot asymmetry test indicated that there was no significant publication bias for sensitivity and specificity in Aβ PET (Egger's test: *p* = 0.89, 0.95) (Figure 7a–b), and similarly, no significant publication bias for sensitivity and specificity was observed for MRI (Egger's test: *p* = 0.79, 0.90) (Figure 7c–d).

## 4 Discussion

The debate around the comparative diagnostic performance of Aβ PET and MRI in AD is a focal point of current research (Wang et al. 2016; Trzepacz et al. 2014; Vandenberghe et al. 2013; Xu et al. 2016). The 2011 NIA‐AA criteria describe Alzheimer's disease as a continuum. Aβ deposition marks the initial stage, followed by neurodegeneration and cognitive decline (Carrillo et al. 2013; Frisoni, O'Brien, and Winblad 2011). On the other hand, MRI captures neurodegeneration, often shown as hippocampal or medial temporal lobe atrophy (Molinder et al. 2021). Recent research has highlighted the effectiveness of Aβ PET imaging (Ruan and Sun 2023; Pemberton et al. 2022). However, the critical question remains as to whether it exceeds MRI in terms of diagnostic accuracy (Vandenberghe et al. 2013). Given the importance of optimizing AD diagnosis and treatment strategies, we performed the first head‐to‐head comparison between Aβ PET and MRI in the diagnosis of AD.

In this meta‐analysis, we pooled the diagnostic performance of Aβ PET and MRI in AD across various comparisons: AD vs. MCI, AD vs. NC, and MCI vs. NC. Our findings reveal similar sensitivities and specificities for both Aβ PET and MRI across these comparisons. Notably, in the 18F‐FMM subgroup, 18F‐FMM demonstrated higher specificity in distinguishing AD from MCI and NC, suggesting its potential as a more precise diagnostic tool in these particular contexts. However, it is important to note that only one study using 18F‐FMM was included, which may have contributed to the differences in specificity due to the limited sample size; further research is required for a definitive conclusion. The similar sensitivities and specificities of Aβ PET and MRI across different comparisons in our study may be due to the fact that both Aβ PET and MRI target different but complementary aspects of AD. While Aβ PET focuses on amyloid‐β deposits, a hallmark of AD, MRI assesses structural brain changes like atrophy, which also correlate with disease progression (Yu, Shan, and Ding 2021; Oldan et al. 2021). Their complementary nature might explain why neither modality significantly outperforms the other in our analysis.

In 2014, a meta‐analysis study by Bloudek et al. (2011) found that MRI's sensitivity and specificity in diagnosing AD from NC were 0.83 and 0.89, respectively, but the study did not include a comparison with Aβ PET. Our analysis addresses this gap, providing a direct comparison between MRI and Aβ PET. Additionally, Ruan and Sun (2023) showed Aβ PET's sensitivity at 0.91 and specificity at 0.81 for AD vs. NC, which is consistent with our findings of 0.93 sensitivity and 0.95 specificity. They also found insignificant differences between 11C‐PIB PET and 18F‐AV45 PET in diagnostic efficacy. Our results corroborate this, showing similar efficacy for 11C‐PIB PET and 18F‐AV45 PET compared to MRI. However, Ruan and Sun (2023) study did not include a comparison with MRI. Our research fills this gap, offering the first comprehensive head‐to‐head comparison between these two important diagnostic tools for AD.

Aβ PET offers high sensitivity in detecting amyloid plaques, a key hallmark of AD, but is costly and less widely available (Brand et al. 2022; Hornberger et al. 2017). MRI, on the other hand, is more accessible and cost‐effective, focusing on brain structure changes (McEvoy and Brewer 2010). However, it may not detect early molecular changes like Aβ PET. Their complementary roles suggest that a combined approach could enhance diagnostic accuracy, but a detailed cost‐effectiveness comparison is needed to fully assess the viability of such integrated diagnostic strategies (Polikar et al. 2010). Atrophy detected by MRI is common in AD but is not specific to the disease. These structural changes can also appear in other neurodegenerative conditions, such as fronto‐temporal or vascular dementia, which limits MRI's diagnostic accuracy (Ortner et al. 2019). In contrast, amyloid PET directly detects AD‐specific pathology, such as amyloid plaques, which are not found in other forms of dementia (Jack, Barrio, and Kepe 2013). The decision on which imaging modality to use in Alzheimer's Disease—Aβ PET or MRI—depends on factors like the clinical situation, availability of technology, and physician preference (van Oostveen and de Lange 2021; Kim et al. 2022). In addition, compared to Aβ PET, FDG PET provides a complementary perspective by capturing functional impairment in the brain, rather than specific amyloid pathology. FDG PET is also more widely available in clinical settings due to its utility in a broader range of diseases, including other forms of dementia (Rabinovici et al. 2011). Each modality has unique advantages, and their selection should be tailored to specific diagnostic needs and resource availability.

In our meta‐analysis, it is crucial to consider limitations in interpreting the results. The heterogeneity of included studies, particularly those by Trzepacz et al. (2014) and Mikhno et al. (2012), was notable. This heterogeneity, likely due to the differences in study design and patient demographics, could have influenced MRI's sensitivities and specificities. A sensitivity analysis indicated these two studies as potential sources of heterogeneity. Furthermore, the meta‐analysis comprised a relatively small sample of just six head‐to‐head comparison studies, which might have led to bias. In addition, another limitation of our meta‐analysis is the “unclear risk” in several studies regarding patient selection, reference standards, and flow and timing. These gaps in reporting may introduce bias and affect the generalizability of our findings. Therefore, larger, well‐structured prospective studies are necessary to corroborate our findings and provide a more definitive conclusion.

## 5 Conclusion

Based on the pooled results, our meta‐analysis indicates that Aβ PET demonstrates similar sensitivity and specificity to MRI in diagnosing Alzheimer's disease. However, the limited number of studies may impact the evidence of the current study; further larger sample prospective research is required to confirm these findings.

## Author Contributions

**Fang Li**: conceptualization, writing–original draft, software, formal analysis. **Jiang Cheng**: software, data curation, formal analysis, writing–original draft. **Kaihui Jin**: software, data curation, formal analysis, writing–original draft. **Li Zhao**: validation, software, writing–original draft. **Junyong Li**: writing–original draft, software. **Jia Wu**: writing–original draft, software. **Xiaolu Ren**: writing–review & editing, conceptualization, methodology, validation, supervision.

## Ethics Statement

This is a systematic review and meta‐analysis; ethics approval and consent to participate are not applicable.

## Consent

Not applicable. This study does not involve human participants.

## Conflicts of Interest

The authors declare no conflicts of interest.

### Peer Review

The peer review history for this article is available at https://publons.com/publon/10.1002/brb3.70111

## Supporting information




**Supplementary Table 1**. Search strategy in PubMed, Embase, and Web of Science databases.
**Supplementary Figure 1**. Forest plot showing the pooled sensitivity of Aβ PET and MRI in detecting AD from NC. AD, Alzheimer's disease; NC normal cognitive control.
**Supplementary Figure 2**. Forest plot showing the pooled specificity of Aβ PET and MRI in detecting AD from NC. AD, Alzheimer's disease; NC normal cognitive control.
**Supplementary Figure 3**. Forest plot showing the pooled sensitivity of Aβ PET and MRI in detecting MCI from NC. MCI, mild cognitive impairment; NC normal cognitive control.
**Supplementary Figure 4**. Forest plot showing the pooled specificity of Aβ PET and MRI in detecting MCI from NC. MCI, mild cognitive impairment; NC normal cognitive control.

## Data Availability

The original contributions presented in the study are included in the article. Further inquiries can be directed to the corresponding authors.
