# Supplementary material for: Comparative Diagnostic Performance of Amyloid‐β Positron Emission Tomography and Magnetic Resonance Imaging in Alzheimer's Disease: A Head‐to‐Head Meta‐Analysis
Source: Brain Behav. 2024 Oct 22;14(10):e70111. doi: 10.1002/brb3.70111 (PMC11494400; doi:10.1002/brb3.70111)

Supplementary Table 1 Search strategy in PubMed, Embase, and Web of Science databases.

| Database | Search strategy |
| --- | --- |
| PubMed (1897) | ("Alzheimer Disease"[Mesh] OR "Dementia"[Mesh] OR “Alzheimer”[Title/Abstract] OR “Dementia”[Title/Abstract] OR “mild cognitive impairment”[Title/Abstract] OR “MCI”[Title/Abstract]) AND ("Positron-Emission Tomography"[Mesh] OR “Positron Emission Tomography”[Title/Abstract] OR “PET”[Title/Abstract]) AND ("Amyloid"[Mesh] OR “florbetapir” [Title/Abstract] OR “flutemetamol” [Title/Abstract] OR “florbetaben” [Title/Abstract] OR “AV45”[Title/Abstract] OR “FMM” [Title/Abstract] OR “PIB”[Title/Abstract]) AND ("Magnetic Resonance Imaging"[Mesh] OR "Magnetic Resonance Imaging"[Title/Abstract] OR "Magnetic Resonance "[Title/Abstract] OR “MRI”[Title/Abstract] OR “MR”[Title/Abstract]) |
| Embase (2987) | ('Alzheimer disease'/exp OR 'dementia'/exp OR ‘Alzheimer’:ab,ti OR ‘Dementia’:ab,ti OR ‘mild cognitive impairment’:ab,ti OR ‘MCI’:ab,ti) AND ('positron emission tomography'/exp OR ‘Positron Emission Tomography’:ab,ti OR ‘PET’:ab,ti) AND ('amyloid'/exp OR 'florbetapir f 18'/exp OR 'flutemetamol f 18'/exp OR 'florbetaben'/exp OR ‘AV45’:ab,ti OR ‘FMM’:ab,ti OR ‘PIB’:ab,ti) AND ('MRI scanner'/exp OR ‘Magnetic Resonance Imaging’:ab,ti OR ‘Magnetic Resonance’:ab,ti OR ‘MRI’:ab,ti OR ‘MR’:ab,ti) |

Supplementary Figure 1 Forest plot showing the pooled sensitivity of Aβ PET and MRI in detecting AD from NC. AD, Alzheimer's disease; NC normal cognitive control.


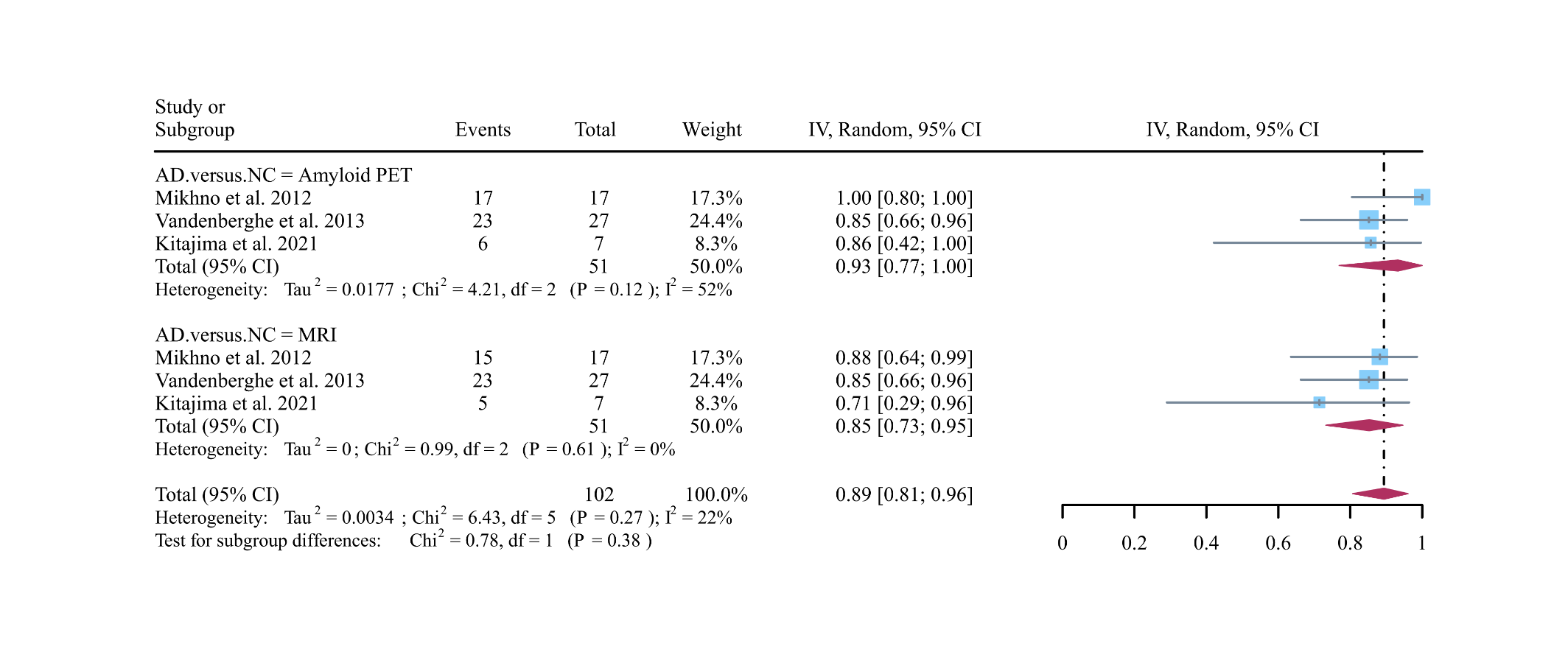


Supplementary Figure 2 Forest plot showing the pooled specificity of Aβ PET and MRI in detecting AD from NC. AD, Alzheimer's disease; NC normal cognitive control.


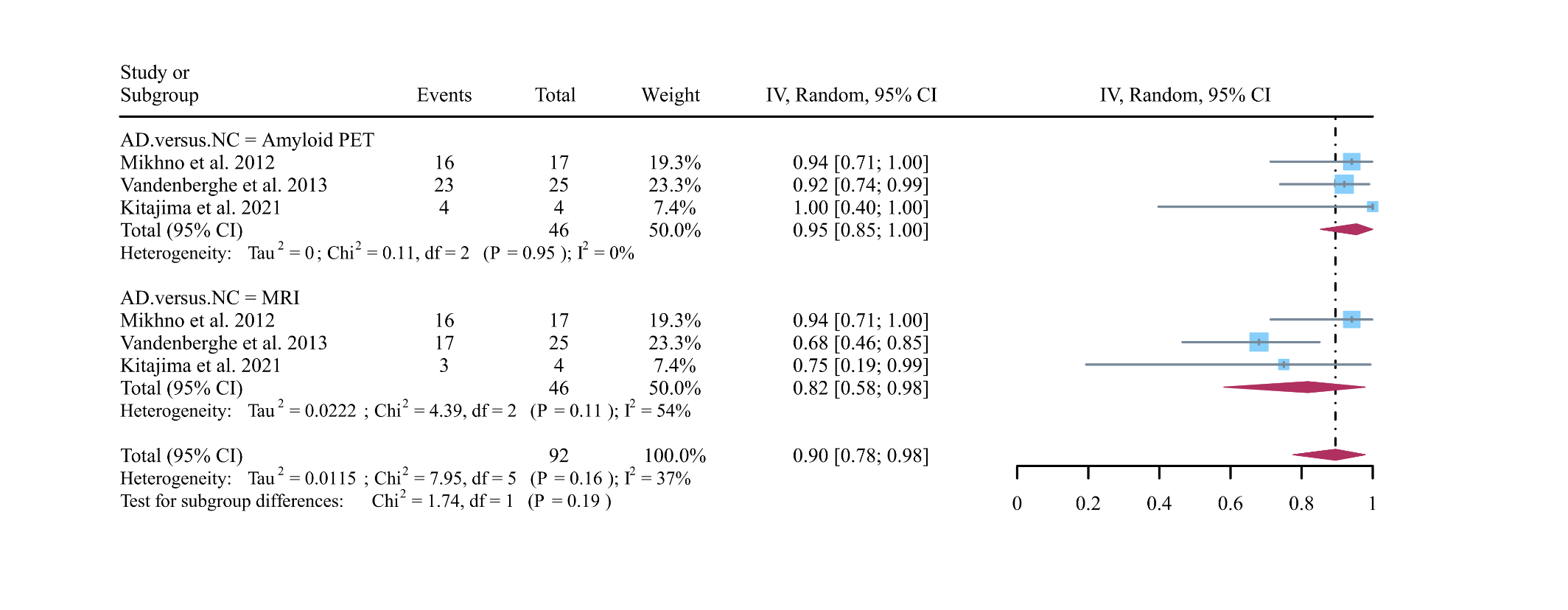


Supplementary Figure 3 Forest plot showing the pooled sensitivity of Aβ PET and MRI in detecting MCI from NC. MCI, mild cognitive impairment; NC normal cognitive control.


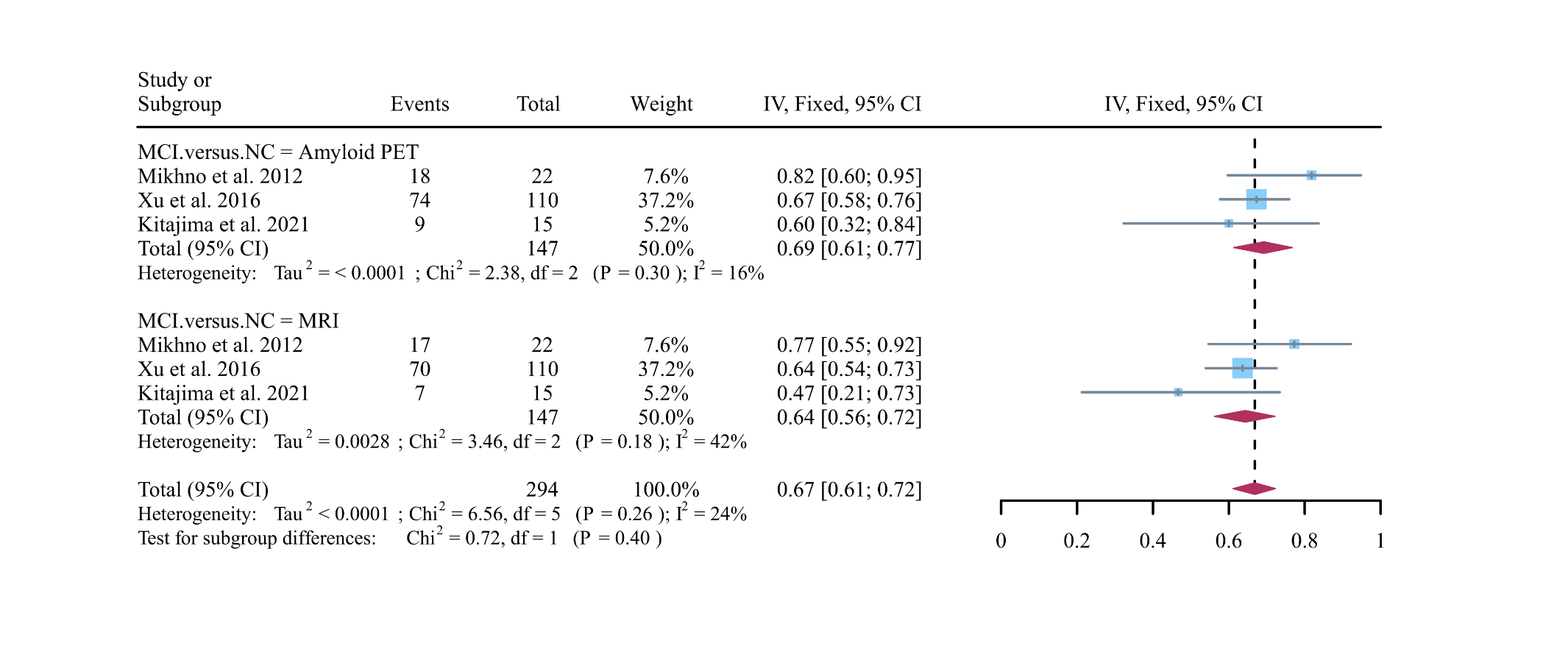


Supplementary Figure 4 Forest plot showing the pooled specificity of Aβ PET and MRI in detecting MCI from NC. MCI, mild cognitive impairment; NC normal cognitive control.


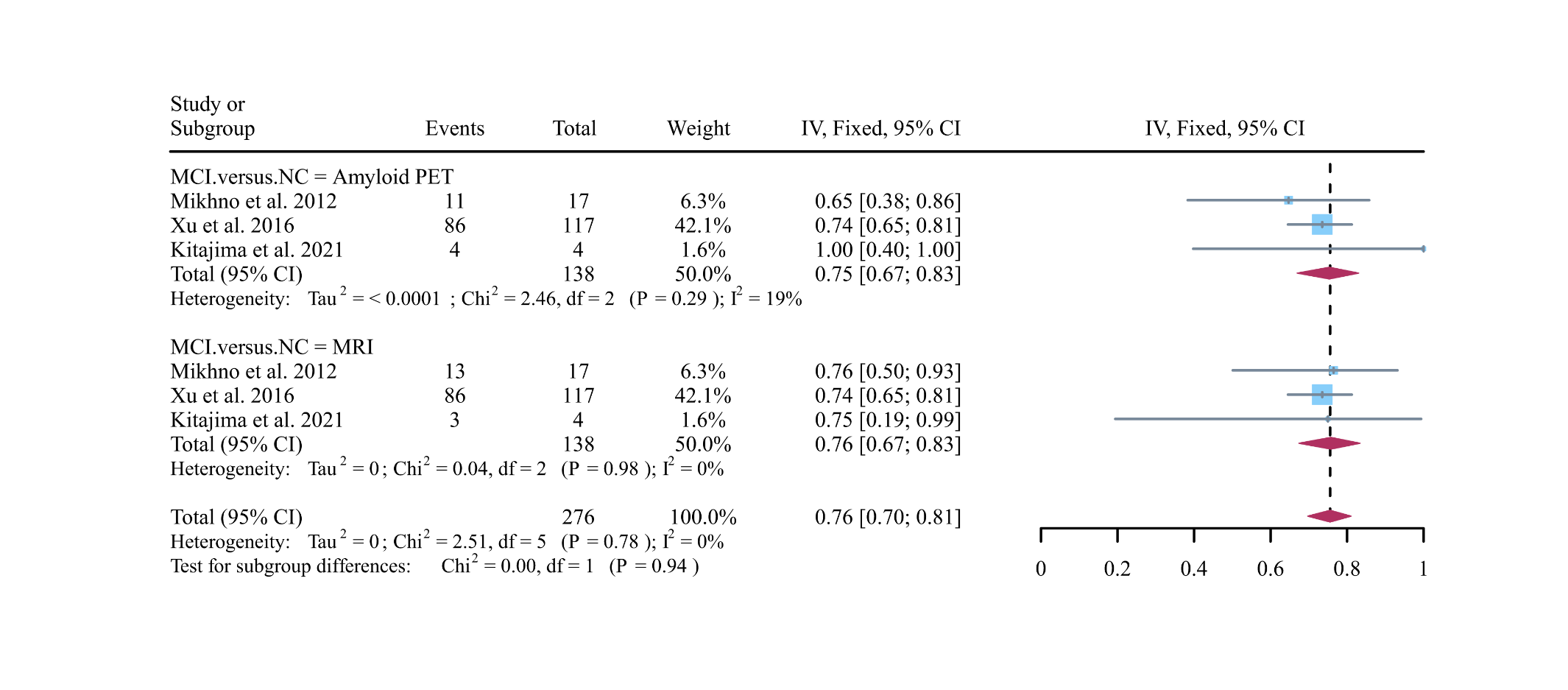

Supplement: Supplementary file 1 — Supplementary Table 1. Search strategy in PubMed, Embase, and Web of Science databases. Supplementary Figure 1. Forest plot showing the pooled sensitivity of Aβ PET and MRI in detecting AD from NC. AD, Alzheimer's disease; NC normal cognitive control. Supplementary Figure 2. Forest plot showing the pooled specificity of Aβ PET and MRI in detecting AD from NC. AD, Alzheimer's disease; NC normal cognitive control. Supplementary Figure 3. Forest plot showing the pooled sensitivity of Aβ PET and MRI in detecting MCI from NC. MCI, mild cognitive impairment; NC normal cognitive control. Supplementary Figure 4. Forest plot showing the pooled specificity of Aβ PET and MRI in detecting MCI from NC. MCI, mild cognitive impairment; NC normal cognitive control. [file BRB3-14-e70111-s001.docx]
